# Revolutionising health and social care: innovative solutions for a brighter tomorrow – a systematic review of the literature

**DOI:** 10.1186/s12913-024-11099-5

**Published:** 2024-07-12

**Authors:** Jennifer Kosiol, Tracey Silvester, Helen Cooper, Stewart Alford, Linda Fraser

**Affiliations:** 1 Health Services Management, Griffith University, Brisbane, Australia; 2 Kaplan Business School, Brisbane, Australia

**Keywords:** Health and social care, Innovation, Sustainability, Organisational culture, Governance

## Abstract

**Background:**

In an era marked by rapid technological advancements, changing demographics, and evolving healthcare needs, the landscape of health services has been undergoing a profound transformation. Innovation has emerged as a central force driving change in the healthcare sector, as stakeholders across the globe strive to enhance the quality, accessibility, and efficiency of healthcare services.

**Objective:**

Within this dynamic context, this systematic literature review explored the barriers and driving forces behind successful health service innovation.

**Methods:**

A comprehensive systematic literature review was conducted using the Griffith University Library search engine and databases that included PubMed, ProQuest, Web of Science, Scopus, and CINHAL. To achieve the study goal, the Preferred Reporting Items for Systematic Reviews and Meta-Analyses guidelines and the associated PRISMA checklist guided the review and reporting method.

**Results:**

Findings from this review identified a need for a universal definition of health innovation that encompasses the unique complexities and challenges within this context. In our comprehensive analysis of healthcare innovation, we have uncovered pivotal findings that underscore the indispensable nature of a well-structured framework.

**Conclusions:**

To succeed in fostering innovation within the health and social care sectors, it is imperative to establish an overarching organisational culture that meticulously addresses the following key components: team challenges; communication and collaboration; governance goals and authentic leadership, environmental engagement; and innovation endurance. Through systematic analysis of existing literature, this review offers a definition of health innovation, covering its conceptual foundations, determinants, and barriers, and provides a framework for creating an innovative culture.

## Background

Healthcare innovation constitutes a multifaceted and dynamic synthesis of technological advancements, research, and the evolution of healthcare delivery systems, to stimulate a transformative shift in patient care paradigms and health management practices [1]. It represents an interdisciplinary venture that amalgamates cutting-edge scientific discoveries, digital technology breakthroughs, and their pragmatic deployment to fundamentally alter the provision and reception of healthcare services [1]. Central to the ethos of healthcare innovation is the aspiration to improve patient outcomes, expand access to high-quality care, and enhance the operational efficiency of healthcare infrastructures [2]. Through the integration of innovative medical devices, the application of artificial intelligence, and the adoption of novel healthcare delivery models, innovation aims to address intricate health dilemmas and meet the bespoke needs of individuals [1, 2]. This progressive orientation not only heralds the advent of pioneering therapeutic interventions and preventive strategies but also recalibrates the healthcare ecosystem to be more adaptive, equitable, and sustainable. Viewed through the lens of innovation, healthcare is about more than just treating illnesses. It’s about reimagining what it means to be healthy and pushing the boundaries to improve population health and well-being [1].

Notwithstanding that while innovation involves creatively considering all aspects of healthcare service and delivery, it can be problematic [1]. This is because even though an innovation might be more effective, efficient, and have better patient outcomes, the implementation is inherently risky and is often targeted at the wrong populations making it unsuitable or unaffordable for the health system [1, 3]. Even when there is strong evidence supporting the advantages of a new technology, its integration can be difficult resulting in uneven adoption and disparities in accessibility across different populations [1]. Implementing healthcare innovations presents challenges such as resistance to change from healthcare professionals and organisations, limited resources, the complexity of interventions, organisational culture, communication barriers, inadequate stakeholder involvement, sustainability concerns, and external influences [3]. Overcoming these difficulties requires careful planning, stakeholder engagement, effective communication, and a focus on sustainability to increase the likelihood of successful implementation and ultimately improve patient care outcomes [4].

The World Health Organisation (WHO) defines health innovation as novel approaches accelerating positive health impact [5]. Applying this definition to a health services management (HSM) context requires understanding the enablers and barriers to successful innovation. Success or failure in innovation depends on various factors, including the innovation itself, the environment, context, and behavioural enablers [6, 7]. This is equally relevant to the health and social care setting, however, in this complex and dynamic environment, there are unique and competing challenges to successful innovation.

Across the research there is limited consensus on the definition of healthcare innovation [8–11]. There is a general presumption that this definition is well known, however, the array of definitions is heavily influenced by particular contexts such as business, health, product development and entrepreneurship. These definitions have fluctuated from a focus on a ‘novel product or technology’ [10], to ‘Ideas’ [9, 11], social issues [12, 13], and more recently ‘a process of change’ [8]. Agreement on a universal definition remains elusive, yet in healthcare’s dynamic and complex environment, a unified definition is imperative, focusing on efficiency, health outcomes, cost-effectiveness, and user experiences unique to health and social care.

In 2011 Dixon-Woods and colleagues raised the paradoxes hindering or supporting healthcare innovation noting varying diffusion rates, and the challenge of participatory and cooperative approaches [14]. Since 2019, emphasis has shifted towards enabling effective and sustainable mechanisms for innovation [15, 16]. It is therefore imperative for health service managers to adopt and integrate successful innovations to overcome barriers and leverage driving forces [17].

Health innovation promises to enhance healthcare and improve outcomes, but implementation is intricate due to the challenges of healthcare performance. Nurturing creativity and novel ideas requires robust leadership [18]. Existing research explores isolated factors of barriers and facilitators, such as organisational culture, financial implications, and sustainability [19, 20]. However, understanding the relationships between these factors in a health services context is essential, particularly regarding interventions that foster a culture of successful and sustainable innovation [20].

Often emerging from the need for improvement, innovations encounter resource-related obstacles. Financial constraints hinder progress, with financial controllers overlooking potential cost savings and efficiency gains identified at the operational level [17, 21]. Health services managers can offer strategic support but are frequently excluded from the decision-making process, leading to covert entrepreneurship and missed opportunities for broader improvement [17, 21, 22]. Sustainability, both practical and financial, is challenging in healthcare, where innovations must be viable [23]. Investment in the cultivation and development of innovation in health services can be disrupted without a clear understanding of the barriers and facilitators for success [24–26].

Despite the multifaceted nature of healthcare innovation, incorporating technological advancements, research, and the evolution of healthcare delivery systems, the literature lacks a unified definition of healthcare innovation. This lack of consensus on definition, influenced by varying contexts such as business, health, product development, and entrepreneurship, points to the necessity of establishing a universal understanding that addresses efficiency, health outcomes, cost-effectiveness, and user experiences unique to health and social care. While existing research has explored isolated factors that act as barriers or facilitators to healthcare innovation, such as organisational culture, financial implications, and sustainability, there is an evident gap in understanding how these factors interact within the health services context. The complexity of implementing healthcare innovations, highlighted by challenges such as resistance from healthcare professionals, limited resources, and sustainability concerns, underscores the need for comprehensive studies that examine the relationships between these factors. The literature on healthcare innovation is missing a detailed exploration of the specific factors in the health context that either support or inhibit the cultivation of successful innovation. Addressing this gap requires a systematic analysis and synthesis of existing literature to illuminate the path forward for healthcare stakeholders, informing evidence-based decision-making and underscoring the process for implementing and sustaining innovations. This calls for research that not only identifies barriers and facilitators but also delves into the intricate relationships between these factors in the health services context, thereby providing a roadmap for fostering innovation that can significantly enhance healthcare delivery and patient outcomes.

The urgency of this review lies in its potential to illuminate the path forward for healthcare stakeholders, inform evidence-based decision-making, and underscore the process for implementing and sustaining innovations. Through systematic analysis and synthesis of existing literature, we aim to provide a comprehensive overview of health services innovation, including its conceptual foundations, determinants, barriers, and impact on health outcomes and system performance.

To address this research gap, our review question was formulated using the ECLIPSE framework which involves a structure approach that caters specifically to questions within health and social care sectors [27]. ECLISPE (Expectation, Client group, Location, Impact, Professionals, Service) [27] guided the review question development with consideration given to various aspects of health innovation such as the expectations/outcomes being sought, the specific population or client group, the setting or location of the study, the type of impact, the professionals involved in the health innovation and the nature of the healthcare service being evaluated [27] See Table 1.

**Table 1:** ECLIPSE framework: review question development

Expectation (E)	Understanding the key factors that drive successful innovation adoption and sustainability
Client group (C):	Health and social care organisations and their stakeholders, including patients, healthcare professionals and administrators
Location (L	Diverse health and social care settings, ranging from hospitals and clinics to community-based care and social services.
Impact (I):	The effectiveness of innovations in improving patient outcomes, enhancing service delivery and achieving organisational goals
Professionals (P):	A range of healthcare providers including doctors, nurses, social workers and management staff involved in the innovation process
Service (SE):	Various types of health and social care services that are potential targets for innovation, including digital health technologies, new care models, and service delivery processes.

Based on the ECLIPSE framework the following research question was formulated:

*What factors facilitate or inhibit the successful adoption, implementation, and sustainability of innovation across diverse health and social care contexts, and how do these factors impact the expectation of client groups, the roles of professionals, and the effectiveness of services?*

This question aims to explore the multi-dimensional aspects of innovation in health and social care, considering the expectations for success, the specific needs and characteristics of different client groups, the settings in which innovations are deployed, the outcomes that are sought, the professionals involved in implementing changes, and the types of services affected by innovation.

## Method

This qualitative review of the literature was completed using the Preferred Reporting Items for Systematic Reviews and Meta-Analyses (PRISMA) guidelines and the associated PRISMA checklist guided the review and reporting method [28]. The authors used the computer application Covidence© as a platform to support the organisation, extraction and review of articles returned from the search strings.

### Search strategy

A comprehensive search was conducted using the Griffith University Library search engine and databases that included PubMed, ProQuest, Web of Science, Scopus and CINHAL. The researchers engaged a library scientist to guide the search strategy. The search included studies published from 01/01/2018 to 18/03/2023. Search strings included a combination of keywords using Boolean operators and truncation (*) where necessary. The following keyword combinations were used as search strings across the databases in Table 2 below:

**Table 2:** Search Strings with Number of Records Returned

No	Search String	Records Returned	Scopus	Web of Science	Medline	ProQuest
11	health W/2 (service* OR care* OR system*) AND Innovation*	886	333	264	182	107
22	health W/2 (service* OR care* OR system*) AND Innovation* and Success*	9	5	2	2	0
33	health W/2 (service* OR care* OR system*) AND Innovation* and Success* AND Implement*	1	1	0	0	0
44	Health w/2 (service* or care* or system*) AND Innovation* AND Success* w/2 implement*	0	0	0	0	0
	Total	896	339	266	184	107

### Inclusion and exclusion criteria

Only English language articles published between January 2018 and January 2023 were reviewed.

Limiting the literature review to English-language papers enabled a streamlined approach to the research process by focusing on the most widely accessible and frequently cited studies, ensuring efficiency and broad relevance within the global scientific community. Focusing the literature review on the last 5 years of healthcare innovations is justified by the rapid pace of technological advancements, the emergence of new health crises like the COVID-19 pandemic, evolving healthcare policies and regulations, shifts in patient expectations towards digital and personalised care, and the need to evaluate the effectiveness and implementation of recent innovations. This timeframe ensures the review captures the most current insights, reflecting the latest in medical technology, patient care strategies, and global healthcare trends. By doing so, it aligns the review’s findings with the current healthcare priorities, regulatory environments, and the latest evidence on innovation effectiveness, making it highly relevant and valuable for informing future healthcare decisions, policymaking, and practice improvements.

The search focused on academic, peer-reviewed materials with full online text, excluding grey literature encompassing, theses, commercially published documents such as technical reports, white papers, and conference proceedings to ensure the highest levels of methodological rigor, reliability and validity in the evidence being reviewed. Included articles were empirical studies relevant to enablers and barriers, including process innovation, in health care services. Articles were excluded if they were existing systematic reviews of the literature as the research question was seeking empirical studies of healthcare innovation.

### Study selection

The authors used a dual independent review of search results; titles and abstracts were screened independently by teams of two reviewers to identify studies that met eligibility criteria. Full-text articles that met the inclusion criteria were further reviewed by all review team members independently and then as a group. Divergences were resolved through discussion with all reviewers until consensus was achieved.

### Quality assessment

We used the Mixed Methods Appraisal Tool (MMAT) 2018 [29] for quality assessment of the research reports and evidence-based articles. MMAT is designed for critical appraisal in mixed methods study reviews and evaluates the methodological quality of qualitative research, randomised controlled trials, non-randomised studies, quantitative descriptive studies, and mixed methods studies. Although not all categories were applicable to our included studies, they were assessed using MMAT, as shown in Table 3. In conducting the MMAT assessment, the researchers agreed that only those papers that scored a “yes” response to questions S1 and S2 of the tool would progress further. In addition, only those papers that scored a “yes” response to 4 or more of the quality criteria questions in the selected methodology were included for data extraction.

**Table 3:**
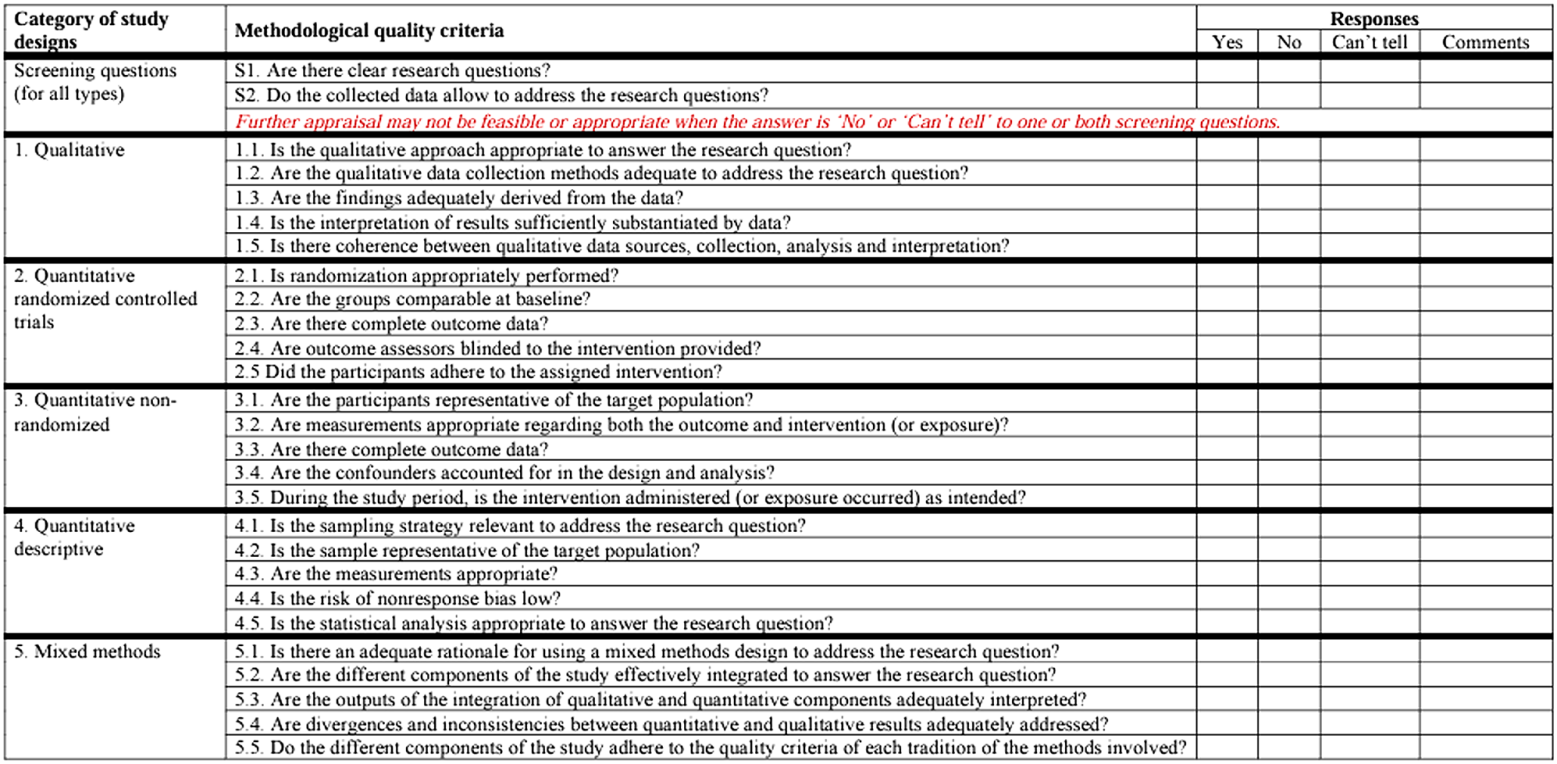
MMAT quality assessment tool [29]

### Data extraction

The following information was extracted from each paper: author, year, country, study name, an overview of the healthcare innovation, barriers, enablers and outcomes, including sustainability of the innovation.

### Data analysis

Using the research question as a guide, key themes were derived from the extracted data by identifying common themes and concepts across the literature. Converging the qualitative and quantitative evidence involved integrating the findings from both types of studies to provide a comprehensive understanding of healthcare innovation [30]. This included extracting key findings from qualitative and quantitative studies; comparing and contrasting the findings to identify similarities, differences and areas of convergence or divergence; and synthesising the evidence using the thematic analysis process guided by Braun and Clarke’s 6-phase guide [31]. Meaningful conclusions were drawn from the convergence that identified implications for practice or policy and highlighted any gaps or inconsistencies in the literature. Findings were aligned with the objectives and research question of the systematic literature review.

## Results

**Fig. 1:**
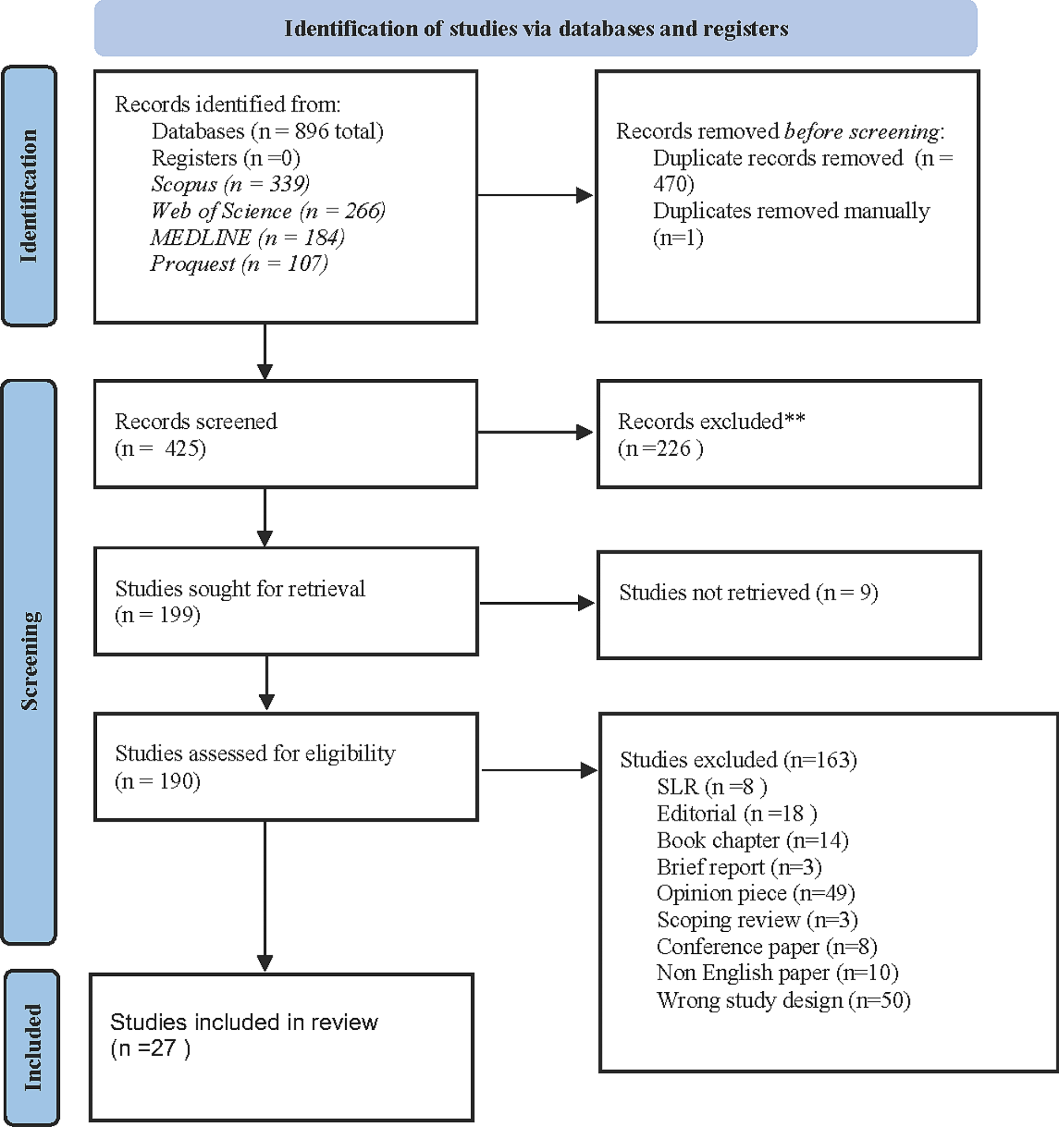
Selection of papers

Figure 1 below shows the selection process using PRISMA [32].

### Study design and location

The twenty-seven (27) included studies were conducted in several countries including United Kingdom (6), Canada (5), United States (3), Germany (3), Brazil (1), South Africa (2), France (1), Indonesia (1), Taiwan (1), Norway (1), Finland (1), Belgium (1), Australia (1). Table 4. shows the included papers, study aims, location of study, year of publication, the MMAT result and the identified themes: (1) Information sharing and helping behaviours; (2) Team specific challenges; (3) Sustainability and diffusion; (4) Governance; (5) Culture; (6) Environmental; (7) Technology; and (8) Definition.

**Table 4:** Selected Papers

Author/s	Year	Title	Aim of the study	Country	Main themes of the paper	MMAT score
Afraz, F.C., Vogel, A., Dreher, C., Berghofer, A.	2021	Promoting Integrated Care through a Global Treatment Budget A Qualitative Study in German Mental Health Care using Rogers’ Diffusion of Innovation Theory	To identify inhibiting and facilitating factors for innovation diffusion.	Germany	1, 2, 3, 4, 5, 6, 7.	5
Aminullah, E., Erman, E.	2021	Policy innovation and emergence of innovative health technology: The system dynamics modelling of early COVID-19 handling in Indonesia	Computer simulation of COVID-19 handling to explain the policy innovation effectiveness.	Indonesia	4, 7.	5
Atkinson, M. K., Singer, S. J.	2021	Managing Organizational Constraints in Innovation Teams: A Qualitative Study Across Four Health Systems	How interdisciplinary teams developing healthcare innovations manage organisational challenges.	United States	1, 2, 3, 4, 5, 6, 7.	5
Barber, S., French, C., Matthews, R., Lovett, D., Rollinson, T., Husson, F., Turley, M., Reed, J.	2019	The role of patients and carers in diffusing a health-care innovation: A case study of “My Medication Passport”	How diffusion of an innovation, My Medication Passport, occurred and roles played by patients.	United Kingdom	1, 2, 3. 4. 5.	5
Bensliman, R., Callorda Fossati, E., Casini, A., Degavre, F., Mahieu, C.	2021	How local stakeholders’ social representations shape the future of ageing in place: Insights from ‘health and care social innovations’ in Wallonia (Belgium)	Explored social representations underlying health and social care innovations	Belgium	3, 4, 5.	4
Brooke-Sumner, C., Petersen-Williams, P., Kruger, J., Mahomed, H., Myers, B.	2019	‘Doing more with Less’: A qualitative investigation of perceptions of South African health service managers on implementation of health innovations	Identified perceptions of constraints to innovation and emergent responsive behaviours.	South Africa	1, 2, 3, 4, 5.	5
Camargo Jr, A. S.	2021	Outpatient regulation system in health management: economic benefits of technological innovations	Evaluated the economic benefits of managing an outpatient appointments system with innovative technology	Brazil	7.	4
Esdar, M., Hübner, U., Thye, J., Babitsch, B., Liebe, J. D.	2021	The effect of innovation capabilities of health care organizations on the quality of health information technology: Model development with cross-sectional data	How core constructs of organizational innovation capabilities are linked to Health Information Technology.	Germany	1, 2, 3, 4, 5, 7.	4
Farmer, J., Carlisle, K., Dickson-Swift, V., Teasdale, S., Kenny, A., Taylor, J., Croker, F., Marini, K., Gussy, M.	2018	Applying social innovation theory to examine how community co-designed health services develop: Using a case study approach and mixed methods	How innovations emerge, develop and are sustained by examining co-designed initiatives through the lens of social innovation.	Australia	1, 2, 3, 4, 5, 8.	5
Greszczuk, C., Mughal, F., Mathew, R., Rashid, A.	2017	Peer influence as a driver of technological innovation in the UK National Health Service: A qualitative study of clinicians’ experiences and attitudes	UK clinicians’ lived experiences of, and attitudes towards, clinical peers endorsing healthcare innovations	UK	1, 2, 4, 5, 8.	4
Khodadad-Saryazdi, A.	2021	Exploring the telemedicine implementation challenges through the process innovation approach: A case study research in the French healthcare sector	Understanding telemedicine in France from an exploratory perspective.	France	1, 2, 3, 4, 5, 7.	5
Leedham-Green, K., Knight, A., Reedy, G. B.	2021	Success and limiting factors in health service innovation: A theory-generating mixed methods evaluation of UK projects	Success and limiting factors in UK health service innovation	UK	1, 2, 3, 4, 8.	5
Lehoux, P., Silva, H. P., Rocha de Oliveira, R., Sabio, R. P., Malas, K.	2022	Responsible innovation in health and health system sustainability: Insights from health innovators’ views and practices	How to influence how health innovators define what problems their solution should tackle and how it may contribute to health systems	Canada	2, 3, 4, 5, 7.	4
Luengen, M., Garrelfs, C., Schulz, C.	2020	Employees’ Acceptance of Health Care Service Innovations: A Study in the Field of Tele-Audiology	The moderating role of organisational support to weaken the negative effects of disruptiveness of innovations through a greater information exchange.	Germany	1, 2, 3, 4, 5, 6.	4
MacGregor, H., McKenzie, A., Jacobs, T., Ullauri, A.	2018	Scaling up ART adherence clubs in the public sector health system in the Western Cape, South Africa: a study of the institutionalisation of a pilot innovation	Analysis of planned organisational change through innovation and challenges associated with innovation.	South Africa	3, 4, 5, 7.	4
McCarthy, A., McMeekin, P., Haining, S., Bainbridge, L., Laing, C., Gray, J.	2019	Rapid evaluation for health and social care innovations: Challenges for “quick wins” using interrupted time series	The aim is to compare two evaluations of the Enhanced Health in Care Homes (EHCH) vanguard, and to investigate the implications of the use of rapid evaluations using interrupted time series (ITS) methods	UK	1, 2, 4.	5
Moser, K. S., Dawson, J. F., West, M. A.	2017	Antecedents of team innovation in health care teams	Explored team-level motivations and how a prosocial team environment may foster innovation.	UK	1, 2, 5, 8.	5
Ploeg, J., Wong, S. T., Hassani, K., Yous, M. L., Fortin, M., Kendall, C., Liddy, C.,Markle-Reid, M., Petrovic, B., Dionne, E., Scott, C. M., Wodchis, W. P.	2019	Contextual factors influencing the implementation of innovations in community-based primary health care: the experience of 12 Canadian research teams	Contextual factors that impacted the implementation of community-based primary health care (CBPHC) innovations and strategies used to address contextual factors influencing implementation of CBPHC innovations.	Canada	1, 2, 3, 4, 5, 6, 7, 8.	5
Rivard, L., Lehoux, P., Miller, F. A.	2019	Double burden or single duty to care? Health innovators’ perspectives on environmental considerations in health innovation design	How those who design new health technologies (devices, technical aids and information technologies) perceive and address environmental considerations in their practice	Canada	1, 2, 3, 4, 5, 6.	4
Saidi, T., Thune, T. M., Bugge, M.	2021	Making ‘hidden innovation’ visible? A case study of an innovation management system in health care	Analysis of the development of an innovation monitoring and management system in the Norwegian health care sector.	Norway	7.	5
Stolldorf, D.P., Havens, D.S., Jones, C.B.	2017	Sustaining innovations in complex health care environments: A multiple-case study of rapid response teams	Examined factors that do and do not support the sustainability of Rapid Response Teams.	USA	1, 2, 3, 4, 5.	5
Torvinen, H., Jansson, K.	2022	Public health care innovation lab tackling the barriers of public sector innovation	The study’s objective is to support especially public organizations in either setting up similar PSI labs or other collaborative innovation policies	Finland	1, 2, 3, 4, 5, 6, 7.	5
Turner, S., D´Lima, D., Sheringham, J., Swart, N., Hudson, E., Morris, S., Fulop, N. J.	2021	Evidence use as sociometrical practice? A qualitative study of decision-making on introducing service innovations in health care	The authors identify three sociometrical mechanisms through which evidence and context shape each other in decision-making: connecting, ordering, resisting.	United Kingdom	1, 2, 6.	4
Urquhart, R., Kendell, C., Folkes, A., Reiman, T.,Grunfeld, E., Porter, G. A.	2018	Making It Happen: Middle Managers’ Roles in Innovation Implementation in Health Care	To empirically examine the role of middle managers relevant to innovation implementation and how middle managers experience the implementation process.	Canada	1, 2, 3, 5, 6, 8.	4
Urquhart, R., Kendell, C., Cornelissen, E., Madden, L. L.,Powell, B. J., Kissmann, G., Richmond, S. A., Willis, C., Bender, J. L.	2020	Defining sustainability in practice: Views from implementing real-world innovations in health care	This study sought to identify how individuals who implement and/or sustain evidence-informed innovations in health care define sustainability.	Canada	1, 2, 3, 4, 5, 6, 7, 8.	5
Wu, J. H., Lin, L. M., Rai, A., Chen, Y. C.	2022	How health care delivery organizations can exploit eHealth innovations: An integrated absorptive capacity and IT governance explanation	How exploitation of eHealth innovations is influenced by its synergistic capacity and how mechanisms governing coordination between IT and non-IT resources can enable the exploitation of eHealth innovations.	Taiwan	1, 2, 3, 4, 5, 6, 7.	4
Zuber, C. D., Moody, L.	2018	Creativity and Innovation in Health Care: Tapping into Organizational Enablers Through Human-Centered Design	Identified enabling conditions that support frontline nurses in their attempts to behave as champions of innovation and change.	USA	1, 2, 3, 4, 5, 6, 7.	5

### Climate of culture

In healthcare innovation, organisational culture profoundly impacts creativity, collaboration, and successful idea implementation [33–37]. Organisational culture, particularly its hierarchical aspects, can hinder innovation, as centralised decision-making obstructs communication and consultation, necessitating a bottom-up approach [38].

The intricate relationship between culture and innovation in healthcare is evident across various dimensions. Whether shaping teamwork dynamics, influencing innovation survival, or steering the implementation and sustainability of innovative practices, culture emerges as a significant determinant [39]. Organisational culture can either foster experimentation and learning or obstruct innovation due to risk-averse norms and hierarchy [35]. To harness the transformative potential of innovation in healthcare, stakeholders must foster a conducive environment that encourages creativity, experimentation, and a contribution to the evidence base, while navigating the challenges posed by existing norms, hierarchies and bureaucracy [40, 41]. The relationship between culture and innovation influences adoption, implementation, and sustainability of innovative approaches [42]. Adapting innovations to fit within specific cultural contexts is a critical step that significantly influences their design, development, implementation, and overall effectiveness. This process ensures that innovations are not only technically sound but also culturally relevant, enhancing their acceptance and utility among target populations. By considering the unique values, beliefs, behaviours, and social norms of different cultures, innovators can create solutions that are more likely to be embraced and integrated into daily practices. This tailored approach to innovation can lead to improved outcomes, as it facilitates a deeper understanding and engagement with the intended users, thereby increasing the likelihood of successful adoption and sustained use. Moreover, by aligning innovations with the cultural context, barriers to implementation are minimised, making the innovation more effective in addressing the specific needs and challenges of the community it is designed for [39, 43]. . Understanding these nuances and tailoring interventions accordingly can bridge the gap between innovative concepts and the communities they aim to serve.

### Team challenges

In a culture that promotes mutual support, information sharing, and community, team-level innovation flourishes [33, 44]. Psychological safety, a key factor, encourages idea sharing and open discussions, fostering trust and cooperation [44]. Organisations benefiting from an environment where ideas can be voiced safely exhibit greater risk-taking and resilience. Hence, cultivating a culture that encourages open communication, supports experimentation, and embraces failure is paramount.

An innovative team culture facilitates diverse perspectives, encouraging novel solutions and mitigating fear of repercussions for unconventional ideas [17, 35, 37, 45]. Healthcare teams often encounter challenges in garnering support for innovations, requiring partnerships and advocacy beyond their immediate community [34, 35, 38, 44, 46–49]. Navigating the delicate balance between novelty and established practices becomes a crucial challenge.

Teamwork and collaboration play a crucial role in driving innovation, especially within a prosocial setting that prioritises mutual support and the sharing of information among its member [50]. However, the deeply ingrained conservative technical culture within healthcare, which requires rigorous empirical evidence to demonstrate cost-effectiveness, can be a significant obstacle to the implementation of new ideas. The conventional norms and practices prevalent in healthcare often pose challenges to the adoption and longevity of ‘innovative concepts,’ potentially stifling their development and integration into the existing system [13, 33, 34, 46, 50, 51].

Healthcare innovation necessitates collaboration across disciplines, but multidisciplinary teams may face challenges like language barriers and a lack of trust and respect among members [36, 40, 44, 46]. Enhancing collaboration and bridging disciplinary boundaries requires fostering open communication, establishing shared goals, and building trust.

### Communication and collaboration

Promoting innovation in healthcare teams relies on information sharing and supportive behaviours [34, 36, 37, 40, 43, 44, 46, 51–54]. This collaborative approach turns potential barriers into opportunities. However, the importance of engaging stakeholders cannot be underestimated as shared expectations and learning among stakeholders are vital features for innovations to extend beyond their initial settings. Individuals acting as boundary spanners, particularly service managers help bridge the gap between innovative ideas and established norms by interpreting and promoting innovations in alignment with strategy and prevailing policy discourses [13].

Information exchange, particularly among professional groups, influences decision-making in innovation strategies. It enhances dynamic innovation and adaptive implementation strategies, creating a protective shield that fosters adoption and diffusion [13, 38, 39, 43, 46]. This convergence in communication drives innovation diffusion, nurturing a culture of innovation within healthcare teams, addressing constraints and fostering collaboration.

Healthcare innovation implementation is a complex journey fraught with challenges, ranging from resource constraints and communication differences to the need for interdisciplinary collaboration [17, 37, 44, 53]. These challenges underscore the importance of effective leadership, open communication, and a supportive environment that encourages experimentation and learning. As healthcare systems continue to evolve, addressing challenges becomes pivotal in ensuring that innovative ideas translate into tangible improvements in patient care and outcomes [34, 36, 38, 51]. By acknowledging and addressing obstacles head-on, healthcare teams can pave the way for transformative innovations that shape the future of healthcare delivery.

### Governance goals and authentic leadership

Governance and policy have emerged as critical determinants in shaping the sustainability, diffusion, and success of innovative health services within the healthcare landscape. Leadership and resource allocation emerge as central facets of governance that influence innovation outcomes [22, 33, 34, 41]. Authentic leadership should encourage shared leadership models, adaptability to change, and a commitment to maintaining necessary resources. Clarity of goals and controlled access to resources are identified as key enabling conditions that facilitate innovative problem-solving [54]. By granting individuals the ability to control specific resources, such as finances, personnel, or time, organisations empower them to devise solutions to challenges. This underlines the importance of governance structures that provide clear directives while allowing for resource autonomy to drive and sustain innovation.

Policy changes and regulatory shifts play a pivotal role in healthcare innovation adoption and sustainability [13, 33, 37, 43, 45, 53]. Navigating these changes, such as shifts in practice standards, requires a delicate balance of understanding and adapting to political structures and regulations. To promote sustainability, innovators and healthcare organisations must remain agile in the face of evolving policies and regulations that affect resource allocation and implementation processes. Policy innovation extends beyond traditional health outcomes to encompass environmental and social considerations [33, 48, 54] highlighting the need for policies that balance patient care and environmental concerns.

Governance and policy serve as bridges connecting the micro-level actions of individuals and teams to macro-level impact [13]. Effective governance structures necessitate engagement with stakeholders at management and policy levels to ensure that innovations are embraced, supported, and integrated into broader healthcare strategies [13]. This integration of stakeholders from meso (management) to macro (policy) levels underscores the importance of a comprehensive governance and authentic leadership approach to innovation diffusion and sustainability.

In the dynamic healthcare innovation landscape, governance and policy shape the adoption, diffusion, and sustainability of innovative health services [13, 33, 35, 36, 38, 39, 44]. By actively aligning innovations with existing regimes, fostering resource control, navigating regulatory changes, promoting an innovation-friendly culture, and addressing societal concerns, effective governance shapes the trajectory of innovation adoption and success [13, 33, 35, 36, 38, 39, 44].

### Environmental engagement

The healthcare environment is a complex interplay of factors including organisational culture, resource availability, and policy, all of which can impact the relevance and success [51]. Implementing innovations successfully demands a keen awareness of these contextual circumstances and the ability to tailor interventions to specific populations. Understanding the geographical, political, and social context is crucial, whether for community-based primary healthcare initiatives or the integration of advanced technologies, as it informs strategies that better meet the diverse communities’ needs [51].

Environmental factors often serve as catalysts for innovation, with rapid technological advancements, evolving reimbursement models, demographic shifts, and changing patient expectations creating a dynamic environment conducive to innovation [34, 41, 55]. These considerations extend beyond initial implementation to the long-term sustainability of healthcare innovations. Integrating environmental considerations early in innovation design can lead to cost savings and broader industry investments in environmentally friendly solutions [38]. A sustainable healthcare industry driven by environmental priorities has far-reaching implications for global population health and health systems.

In healthcare, specific barriers, such as heavy regulation, bureaucracy, and risk-averse attitudes, can hinder innovation. The organisational environment can either facilitate or obstruct innovation. Healthcare organisations fostering an innovation-friendly environment through resource allocation, collaboration promotion, and a culture of experimentation provide fertile ground for innovative ideas to flourish [36]. External stakeholders, including consumers and providers, also wield significant influence over the innovation landscape, highlighting the interconnectedness between the environment and innovation outcomes.

The role of the environment in healthcare innovation cannot be overstated. It encompasses a multitude of factors influencing innovation at every stage, from conception to sustained implementation [49]. Environmental considerations inform strategy, shape decision-making, and strongly influence the success and sustainability of innovations [49]. In the pursuit of meaningful progress in healthcare, stakeholders must recognise and harness the environment’s role as a driving force behind innovation, shaping the future of healthcare delivery and improving consumer outcomes.

### Innovation endurance

The sustainability and diffusion of innovations in healthcare are crucial factors shaping progress, improving consumer outcomes, and revolutionising healthcare [33, 37, 43, 51, 52]. Examining these concepts offers insights into creating lasting impact within the healthcare ecosystem, unveiling the intricate relationship between innovation and healthcare processes.

Innovation sustainability transcends projected cost savings and holds a minor role in enduring innovation. Diffusion, the spread of innovations within a social system, relies on multifaceted factors, including innovation characteristics, communication channels, timing, and social context [33, 37, 43, 51, 52]. Acknowledging these variables and their interconnectedness offers a roadmap for effective diffusion strategies.

A cohesive approach to sustainability and diffusion emerges as indispensable in the implementation of innovations, including groundbreaking initiatives such as telemedicine within healthcare organisations. Ultimately, the exploration of sustainability and diffusion in healthcare demonstrates the profound interdependence between innovation, sustainability, and societal progress [39, 53]. Healthcare organisations, aiming to optimise patient care, enhance efficiency, and innovate, can harness the relationship between sustainability and diffusion to shape a brighter healthcare future.

### Defining health innovation

The definition of health innovation is largely presumed across the studies [13, 33, 46–48, 51] with few studies providing a formal definition [12, 13, 39, 44, 47, 48]. Health innovation encompasses the introduction and implementation of new ideas, processes, products, or technologies in healthcare and related services, extending beyond drugs or medical procedures. It involves creative problem-solving, adapting to societal challenges, and creating socio-political change [33]. Health service innovation aims to enhance healthcare quality, efficiency, and outcomes through evidence-informed interventions and advanced technologies, creating value for stakeholders, consumers, and society. It drives progress, addresses inequalities, and encompasses technological advancements, policy changes, and shifts in practices and behaviours. To ensure that the concept of healthcare innovation remains relevant and effective in addressing the evolving challenges and opportunities within the healthcare sector to ultimately guide research, investment, and policy decisions towards approaches that are technologically advanced, inclusive, patient-centred, and aligned with broader health and societal goals, the authors propose the following comprehensive definition for healthcare innovation:

*“a deliberate and coordinated effort to introduce transformative and sustained changes that enhance health outcomes, organisational efficiency, cost-effectiveness, and user experiences in the healthcare sector”.*

Having a standardised definition of healthcare innovation is crucial for ensuring consistency and clarity across the healthcare ecosystem. It allows policymakers, researchers, healthcare providers, and industry stakeholders to align on objectives, measure progress effectively, and prioritise investments and initiatives that have the greatest potential to improve health outcomes. A standardised definition facilitates the identification and dissemination of effective innovations, encourages collaboration, and helps in setting regulatory and ethical standards. Moreover, it aids in evaluating the impact of new technologies and methodologies on patient care, operational efficiency, and health equity, ensuring that the benefits of innovation are accessible and beneficial to all segments of the population. Having a comprehensive definition of healthcare innovation establishes a common understanding among stakeholders, enabling them to address the complex challenges facing healthcare more effectively today. This shared clarity facilitates the use of an effective framework for creating a culture of innovation, ensuring that efforts are aligned, and resources are optimally utilised to foster sustainable advancements that improve patient care, enhance efficiency, and effectiveness.

## Discussion

### Health innovation framework

**Fig. 2:**
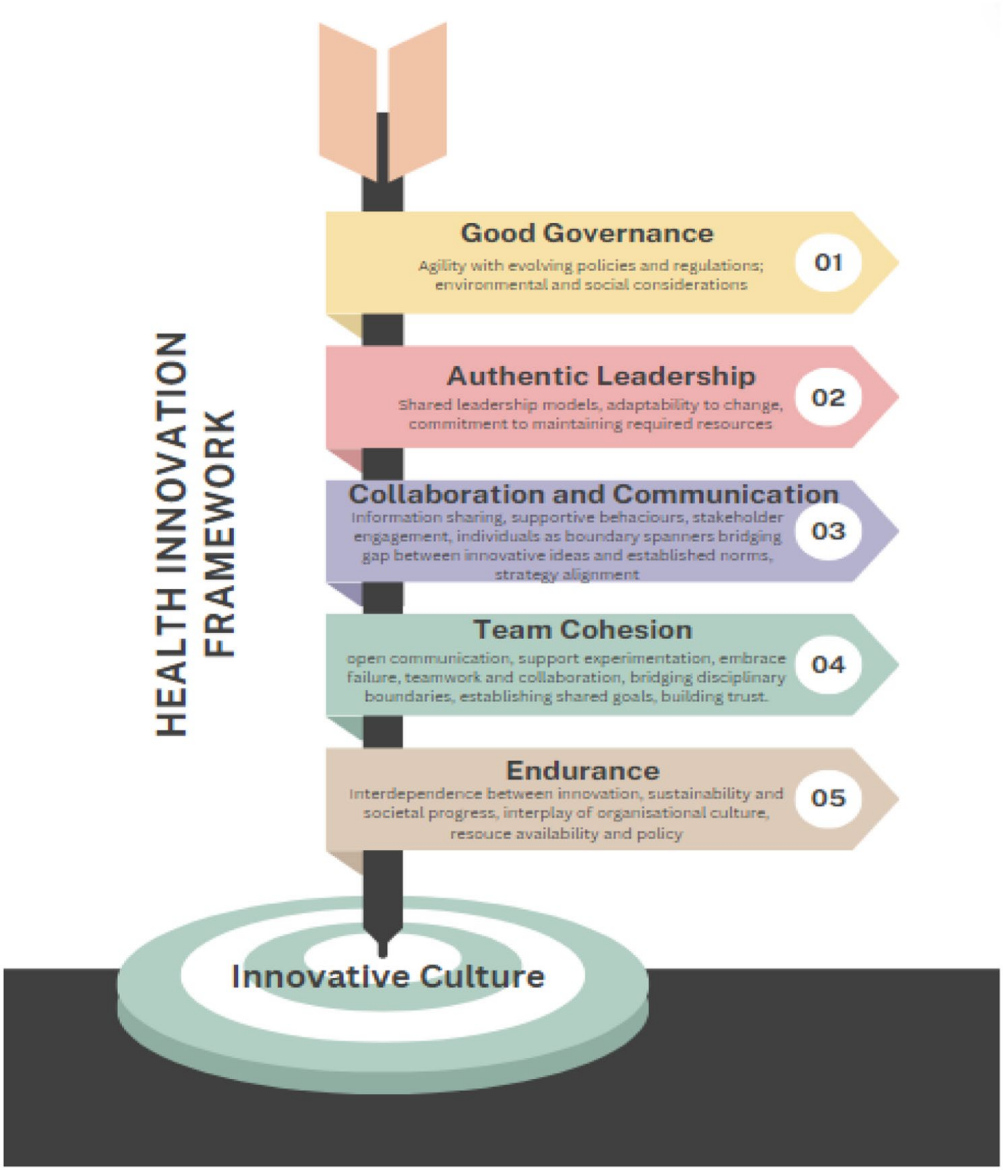
Health Innovation Framework

Organisations approaching innovation and improvement require a progressive framework that is designed to systematically drive an innovative culture informed by the principles of Good Governance, Environmental Engagement, Authentic Leadership, Collaboration and Communication, Team Cohesion, and Endurance. Figure 2. below outlines the principles that are essential to successful health innovation. The overarching principle that underpins this framework is a culture of innovation. The Health Innovation Framework extends beyond traditional innovation implementation frameworks like ReAIM (Reach, Effectiveness, Adoption, Implementation, Maintenance) and the Consolidated Framework for Implementation Research (CFIR), which aim to guide and evaluate innovation and improvement efforts. It emphasises the crucial elements of organisational culture and leadership practices that are fundamental in nurturing and sustaining successful innovation.

The necessity of developing innovation-friendly cultures that foster innovative thinking is grounded in empirical knowledge [56–59]. The health innovation framework offers the principles required for such a culture. The Health Innovation Framework provides the foundation for successful and sustainable healthcare innovation. Good governance establishes ensures innovations are developed and implemented in a responsible, ethical manner, aligned with both organisational goals and regulatory standards. This fosters a stable and trustworthy environment conducive to exploring new ideas. Simultaneously, authentic leadership is crucial as it engenders a culture of trust, openness, and ethical behaviour. Leaders who demonstrate authenticity inspire their teams, encourage the free exchange of ideas, and empower individuals to take initiative, thereby acting as catalysts for innovation.

Moreover, the role of collaboration, communication, team cohesion, and endurance cannot be overstated in the context of healthcare innovation. Effective collaboration and communication across multidisciplinary teams enhance the integration of diverse expertise and perspectives, leading to more creative and comprehensive solutions. A cohesive team environment, marked by mutual support and trust, further encourages the willingness to experiment with and adopt innovative practices. Finally, the resilience to endure through challenges is essential for navigating the inevitable obstacles that arise during the innovation process. Together, these elements create a dynamic framework that supports the continuous flow of innovative ideas and their transformation into practices that significantly improve healthcare delivery and patient outcomes.

The correlation between culture, environment, and healthcare performance underscores culture’s significant influence on innovation. A culture promoting open communication, team collaboration, information sharing, and good governance leads to improved consumer care, operational efficiency, and adaptability to innovation. Among these factors, culture emerges as the most critical determinant of innovation success or failure. Healthcare organizations prioritizing innovation in their cultural values tend to attract individuals passionate about driving positive change and enhancing innovative problem-solving capacity.

### Limitations

While an extensive systematic literature review has been conducted this review has limitations that should be considered. These include a potentially limited scope in terms of the potential publication bias due to excluding grey literature which serves as a crucial counterbalance to publication bias by broadening the spectrum of accessible information. However, the exclusion of grey literature in this review allowed for a rigorous quality assurance, consistency with focusing on established knowledge and consensus within the field, rather than capturing the breadth of ongoing or preliminary research. Additionally, the generalisability of the findings may be limited to the countries included in the review.

## Conclusion

This systematic review sheds light on the critical aspects of health service innovation, emphasising the need for a universal definition and a well-structured framework to foster successful innovation in healthcare settings. In this paper, we make a dual contribution to the field of health innovation. First, we extend the existing body of knowledge by providing new insights and empirical evidence on the mechanisms and outcomes of health innovation practices. Our findings enrich the academic and practical understanding of how innovation can be effectively implemented within healthcare settings. Second, recognising the evolving landscape of healthcare services, we introduce a contemporary definition and framework for healthcare innovation. This framework not only encapsulates the multifaceted nature of innovation in healthcare but also serves as a guide for practitioners and policymakers aiming to foster advancements in healthcare services. By proposing this definition and framework, we aim to set a new direction for future research and practice, enabling healthcare services to adapt and thrive in the face of changing global health challenges.

While the review offers valuable insights into the barriers and driving forces behind health service innovation, it also highlights the complexities and challenges inherent in this dynamic field. There is a wealth of empirical knowledge regarding the necessity for developing innovation friendly cultures that embrace and foster innovative thinking. However, moving forward future research should address the methods of approaching the development of cultural aspects, and successful implementation of innovative ideas that are often subject to failure. Whilst the focus for healthcare delivery remains embedded in economic and fiscal cost savings, innovative concepts that do not demonstrate such savings continue to be accorded low priority. By overcoming these challenges, the healthcare sector can better leverage innovation to enhance quality, accessibility, and efficiency in delivering healthcare services, ultimately contributing to improved health outcomes and system performance.

To further understand how to develop innovation-friendly cultures, studies must focus on strategies not only for innovators but also for policymakers and high-level decision-makers. Converting innovative ideas into practice in healthcare relies heavily on innovators’ ability to garner support in an environment resistant to novel approaches. Examining why some projects fail while others with less potential succeed can inform strategies for achieving desired outcomes.

## Data Availability

No datasets were generated or analysed during the current study.

## Abbreviations

WHO: World Health Organisation
HSM: Health Services Management
PRISMA: Preferred Reporting Items for Systematic Reviews and Meta-Analyses
MMAT: Mixed Methods Assessment Tool
